# Feasibility and Acceptability of a Meditation Mobile App Intervention for Adolescent and Young Adult Survivors of Childhood Cancer

**DOI:** 10.3390/ijerph21050584

**Published:** 2024-05-02

**Authors:** Gary Kwok, Archana Sharma, Ivelisse Mandato, Katie A. Devine

**Affiliations:** 1 Center for Discovery and Innovation, Hackensack Meridian Health, Nutley, NJ 07110, USA; 2 Pediatric Population Science, Outcomes, and Disparities Research Section, Division of Pediatric Hematology/Oncology, Rutgers Cancer Institute of New Jersey, New Brunswick, NJ 08901, USA

**Keywords:** AYA, digital health, mindfulness-based intervention

## Abstract

Background: Adolescent and young adult (AYA) survivors of childhood cancer are increasingly recognized as a vulnerable group with unique emotional, social, and practical needs due to the intersection of cancer survivorship and normal developmental processes. Mindfulness meditation has shown early efficacy in improving psychological distress among cancer patients. However, the overall scientific study of app-based mindfulness-based interventions is still in its early stages. The goal of this study was to evaluate the feasibility and acceptability of a commercially available mindfulness mobile app intervention “Ten Percent Happier” among AYA survivors of childhood cancer. Methods: We conducted a single-arm pilot intervention with 25 AYA survivors of childhood cancer ages 18–29 years. Results: A total of 108 potentially eligible individuals were initially identified for screening. Of the 45 individuals reached (contact rate = 41.67%), 20 declined to participate; 25 were enrolled in the study and completed the baseline survey (enrollment rate = 55.56%). Twenty-one participants completed the study (retention rate = 84%). Changes in several outcomes were promising, with medium to large effect sizes: Mindfulness (*d* = 0.74), Negative Emotion (*d* = 0.48), Perceived Stress (*d* = 0.52), and Mental Health (*d* = 0.45). Furthermore, results suggested that participants with consistent app usage showed greater improvement in reported outcomes than those who stopped their usage (e.g., Mindfulness: *d* = 0.74, Perceived Stress: *d* = 0.83, Mental Health: *d* = 0.51; Meaning and Purpose: *d* = 0.84; and Sleep Disturbance: *d* = 0.81). Qualitative feedback indicated high satisfaction, but participants suggested adding group or individual peer support to improve their experience with the app. Conclusions: AYA survivors can be difficult to reach, but a mindfulness app was feasible and acceptable to this group. In particular, the robust retention rate and high satisfaction ratings indicate that the meditation mobile app was well received. Preliminary results suggest positive changes in health-related quality of life outcomes, warranting a larger efficacy trial.

## 1. Introduction

There are over 500,000 childhood cancer survivors in the United States [1]. Significant progress in treatments for childhood cancer has resulted in an overall survival rate of approximately 84% [2], but survivors are at risk for significant medical (e.g., secondary malignancies) and psychosocial (e.g., anxiety) late effects from treatment [3,4,5]. Adolescent and young adult (AYA) survivors of childhood cancers are a particularly important group to focus on because of their unique emotional, social, and practical needs [6]. The period of young adulthood from ages 18 to 29 years, also termed “emerging adulthood,” is characterized by changes in major life domains, as young adults are tasked with forming their unique identity, pursuing higher education, starting a career, becoming financially independent, forming romantic relationships, and possibly starting a family of their own [7]. Managing cancer survivorship, in addition to these normative tasks, can be challenging. Although a majority of survivors demonstrate resiliency, the data suggest that childhood cancer survivors are at increased risk of poor mental health compared to their non-cancer peers [8] and other risks such as social problems, including lower rates of marriage and independent living, that can cause stress and adversely affect mental health [9]. While 20–25% of AYA survivors of childhood cancer report experiencing impaired mental health [10,11], many more experience subclinical levels of distress. However, most AYA cancer survivors do not receive formal psychological treatment [12,13], suggesting that alternative interventions are needed to reach more survivors.

Complementary and Alternative Medicine (CAM) such as meditation and other mindfulness modalities have become increasingly popular [14] and have shown efficacy in improving psychological distress among adult cancer patients [15]. Although participating in meditation for anxiety and stress reduction is recommended by the Society of Integrative Oncology Clinical Practice Guidelines [16], cancer patients commonly report barriers to practice. For example, it is burdensome to travel to attend face-to-face sessions when enrolled in a class or intervention study [17]. Even when virtually or remotely delivered (e.g., Zoom), there are other barriers to attending mindfulness practice sessions: (1) virtual weekly attendance may not fit participants’ schedule; (2) meditation programs can be very intense (e.g., 90 min sessions, 8–12 weeks long, etc.); and (3) they could be costly if not covered by insurance [18]. Thus, alternative methods to deliver mindfulness interventions should be considered.

Most AYA cancer survivors own a mobile phone, regularly use mobile apps, and are interested in accessing supportive care information via mobile apps [19,20]. Unlike traditional mindfulness interventions, mobile app interventions can be short (e.g., 10 min a day), with the daily goal adjusted to fit one’s schedule [21]. Commercially available mobile apps offer the advantages of being readily available, professionally developed, and maintained to meet the requirements of current operating systems and devices. Mindfulness intervention using mobile delivery has demonstrated early efficacy (e.g., significant positive impact on irritability, affect, and stress) across cancer populations [21,22,23,24]. However, the overall science surrounding online and app-based mindfulness-based intervention is still in its early stages and far from achieving consensus about the efficacy of intervention platforms or understanding the mechanisms of action [22,23,24,25]. Furthermore, unlike traditional mindfulness-based interventions, there are limited data about the clinical benefits of commercially available apps for cancer survivors [26,27]. Studying mobile mediation apps in AYA survivors is particularly important, as digital interventions can overcome geographic and time barriers to accessing intensive in-person or virtual interventions and may be more appealing to this digitally native population [28].

This study aimed to determine the feasibility, acceptability, and usability of “Ten Percent Happier”, a mindfulness mobile app, among AYA survivors of childhood cancer. A secondary aim was to examine changes in mindfulness, perceived stress, emotion, general health status, meaning and purpose, and sleep to describe the preliminary effects of the intervention on key outcomes.

## 2. Materials and Methods

### 2.1. Ethics Approval

This study is approved by the Rutgers Biomedical and Health Sciences Institutional Review Board (IRB# Pro2021000602). All participants provided electronic informed consent prior to their participation in the study. The datasets generated and analyzed during the study are available from the corresponding author upon request.

### 2.2. Study Design

This study was a single-arm feasibility trial with assessments conducted at baseline and postintervention (8 weeks). During the 8-week intervention period, participants were encouraged to use the mindfulness meditation mobile app (i.e., “Ten Percent Happier”) intervention daily. In the first four weeks, research staff would contact participants via text message to check in and answer any questions.

### 2.3. Eligibility Criteria

Participants were eligible if they (1) were 18–29 years old; (2) had a history of cancer and completed treatment at least two years prior to the study start date; (3) were English-speaking; (4) had an email address to register with the app; and (5) had access to a mobile phone with the capability of downloading the study mobile application. Exclusion criteria included anyone who self-reported currently being treated for PTSD, had a history of psychosis or epilepsy/seizures (there is a small likelihood that mindfulness meditation may link to adverse events such as re-experiencing traumatic memories), or had a documented or self-reported cognitive impairment that would prevent the completion of survey measures per the screening questionnaire.

### 2.4. Recruitment

Participants were recruited between September 2021 and August 2022. Potential participants were identified using the electronic medical record review and referrals from treating physicians/nurses/social workers from the local survivorship clinic at the Rutgers Cancer Institute of New Jersey. The study was also advertised to survivors who had completed another study within the clinic and agreed to future contact about research opportunities. The trained research staff contacted potentially eligible patients to assess their interest. Potential participants would complete a brief screening questionnaire to confirm eligibility. Eligible participants were provided with an informed consent document detailing the study expectations, procedures, assessment schedule, and compensation. They were also provided with an opportunity to ask any questions and an electronic informed consent form. Following consent, participants would receive the baseline questionnaire and the instructions on how to download and install the meditation mobile app, Ten Percent Happier.

The goal was to recruit 25 participants for this feasibility study. It is recommended that a sample size be between 20 and 25 for it to be adequate to estimate effect sizes for a single-group pilot study [29]. Power calculations were run using G*Power 3.1 (Heinrich-Heine-Universität Düsseldorf, Düsseldorf, Germany). With a two-sided alpha set at 0.05 and a sample size of 25, we expected to have 80% power to detect a Cohen’s *d* effect size of 0.58. Similar published work on meditation has demonstrated improvements in mindfulness (*d* = 1.11; [30]) and PROMIS (*d* = 0.60 to 0.79; [31]) outcomes. Thus, a sample size of 25 was deemed adequate to address the aims of the study.

### 2.5. Intervention

Per the developers, Ten Percent Happier is “geared towards those who are new to mindfulness or haven’t practiced meditation for a while and need a refresher”. The app offers a beginner meditation course called “The Basics”, featuring seven short (<10 min) sessions. The Ten Percent Happier app also includes many options for guided meditation courses, meditation “singles” (stand-alone meditation sessions), sleep meditations, and podcasts, all in audio and video formats. The podcast hub has four different podcasts as an in-app feature. Each one covers various topics, features special guests such as celebrities, and discusses ways to be more mindful in one’s daily life. To mimic naturalistic use, participants were instructed to use the app however they would like for 8 weeks following enrollment. The Ten Percent Happier memberships were provided to participants for free for the duration of the study.

### 2.6. Measures

Participants received two surveys via REDCap—at baseline (week 0) and postintervention (week 8)—to assess mindfulness, perceived stress, emotion, general health status, meaning and purpose, sleep, and feasibility and acceptability outcomes. Participants were incentivized with a USD 25 Amazon gift card for completing each questionnaire. Demographic and clinical characteristics were collected in the baseline survey.

#### 2.6.1. Feasibility and Acceptability

Feasibility was examined through the study contact, enrollment, and retention rate. Acceptability was examined using the User Version of the Mobile Application Rating Scale (uMARS). uMARS is a 26-item tool to assess the quality of mHealth apps [32]. It yields four subscale mean scores: Engagement, Functionality, Aesthetics, and Information Quality. In addition, it assesses an app’s subjective quality and perceived impact. A total mean score is calculated, reflecting the overall app quality; higher scores indicate a better user experience. In this study, the Cronbach’s alpha coefficient for baseline scores was 0.71 for Engagement, 0.66 for Functionality, 0.69 for Aesthetics, 0.78 for Information, 0.82 for App Subjective Quality, and 0.89 for Perceived Impact. In addition, user data were tracked including minutes and days used. 

All enrolled participants were invited to participate in an exit interview regardless of the extent to which they completed the intervention. Exit interviews focused on participants’ experiences during the study. Participants were asked to (1) identify strengths and weaknesses of the intervention, (2) share their experience with the specific features and components, and solicit new ideas to improve the mobile app (e.g., aesthetic, features that can improve their engagement and/or new features that they would like to see, etc.), and (3) provide feedback on other mindfulness apps in the market. Exit interviews were conducted via Zoom and lasted approximately 20 to 45 min each.

#### 2.6.2. Mindfulness

The Mindful Attention Awareness Scale (MAAS) is a 15-item scale designed to assess mindfulness, defined as a receptive state of mind in which one attends and observes what is occurring in the present without judgment [33]. The response items are rated on a 6-point Likert scale ranging from 1 (almost always) to 6 (always never) A total mean score is computed, with higher scores reflecting greater dispositional mindfulness. In this study, the Cronbach’s alpha coefficient for baseline MAAS scores was 0.82.

#### 2.6.3. Cognitive Reappraisal

We also used the Cognitive Reappraisal subscale from the Emotion Regulation Questionnaire (ERQ) [34] to measure cognitive reappraisal, a pathway through which mindful practice can improve emotion. The response items are rated on a 7-point Likert scale ranging from 1 (strongly disagree) to 7 (strongly agree). A total mean score is calculated, with higher scores reflecting the greater use of reappraisal. In this study, the Cronbach’s alpha coefficient for baseline ERQ–Cognitive Reappraisal scores was 0.88.

#### 2.6.4. Positive and Negative Emotions

Positive and negative emotions were examined using the International Positive and Negative Affect Schedule (PANAS) Short Form (I-PANAS-SF), a 10-item measure that assesses positive and negative emotions [35]. The response items are rated on a 5-point Likert scale ranging from 1 (never) to 5 (never). A total sum score is calculated separately for positive and negative subscales, with higher scores reflecting greater levels of each. In this study, Cronbach’s alpha coefficient for the baseline positive score was 0.69 and for the negative score was 0.67.

#### 2.6.5. Perceived Stress

Perceived Stress Scale (PSS-10) was used to measure the individual’s appraisals of their stress [36]. The response items are rated on a 5-point Likert scale ranging from 0 (never) to 4 (very often). Scores range from 0 to 40 with higher scores indicating higher perceived stress. In this study, Cronbach’s alpha coefficient for baseline PSS scores was 0.81.

#### 2.6.6. Global Health

The Patient-Reported Outcomes Measurement Information System (PROMIS) Global Health was used to measure physical and mental health [37]. The Global Health scale consists of 10 items measuring physical health, physical functioning, general mental health, emotional distress, satisfaction with social activities and relationships, ability to carry out usual social activities and roles, pain, fatigue, and overall quality of life [38]. It consists of two 4-item summary scores: a Global Physical Health (GPH) score and a Global Mental Health (GMH) score. Cronbach’s alpha coefficient of the baseline score was 0.75 for Physical Health and 0.84 for Mental Health.

#### 2.6.7. Meaning and Purpose

The PROMIS Meaning and Purpose short form consists of 4 items used to assess one’s sense of life having purpose and that there are good reasons for living [39]. The response items are rated on a 5-point Likert scale ranging from 1 (not at all) to 5 (very much). Higher scores indicate hopefulness, optimism, goal-directedness, and feelings that one’s life is worthy. Cronbach’s alpha coefficient for the baseline score was 0.94.

#### 2.6.8. Sleep Disturbance 

The PROMIS Sleep Disturbance short form consists of 4 items designed to assess self-reported general sleep and sleep disturbance [40]. The response items are rated on a 5-point Likert scale ranging from 1 (very good/much) to 5 (very poor/not at all). A higher score corresponds to greater sleep disturbance or sleep-related impairment. Cronbach’s alpha coefficient for the baseline score was 0.84.

#### 2.6.9. Exit Interview

All enrolled participants were invited to participate in an exit interview regardless of the extent to which they completed the intervention. Exit interviews focused on participants’ experiences during the study. Participants were asked to identify strengths and weaknesses of the intervention, share their experience with the specific features and components, and solicit new ideas to improve the mobile (e.g., aesthetic, features that can improve their engagement, and/or new features that they would like to see, etc.); they were also asked for demonstrations and comparisons of other mindfulness apps in the market. Within each of these areas of inquiry, specific questions and optional probes were available to the interviewer if needed, based on the participant’s previous responses and to gather additional details. Exit interviews were conducted via Zoom and lasted approximately 20 to 45 min each.

### 2.7. Statistical Analysis

Descriptive analysis was used to describe sample baseline characteristics, feasibility and acceptability, objective user data, and self-reported clinical outcomes. Comparison tests (i.e., paired *t*-tests) were used to examine the change in self-reported clinical outcomes, with Cohen’s *d* calculated for effect sizes. All statistical analyses were performed using Stata (version 14.0; Stata Corp LLC, College Station, TX, USA), with significance inferred at *p* < 0.05.

The interview transcripts were hand-coded line by line to identify possible coding units related to the topics covered in the interview guide. The core themes were identified deductively based on interview domains (e.g., feedback for improvement, app interface comparison, and factors associated with usage).

## 3. Results

### 3.1. Participants’ Characteristics

At baseline, the mean age of the study participants was 23.35 years (*SD* = 3.64). Many of the participants were White (56%), working (64%), and college-educated (48%). A majority of the participants were single (68%), living with their parents (56%), and insured either through their employer or school (52%). Many (40%) of the participants had acute lymphocytic leukemia (ALL) and received chemotherapy (96%) and/or radiation (24%). Table 1 shows the sociodemographic information about the sample.

### 3.2. Feasibility and Acceptability

Feasibility and acceptability were examined through the study contact, enrollment, and retention rates, user data, uMARS, and exit interviews.

#### 3.2.1. Feasibility of Ten Percent Happier

**Figure 1:**
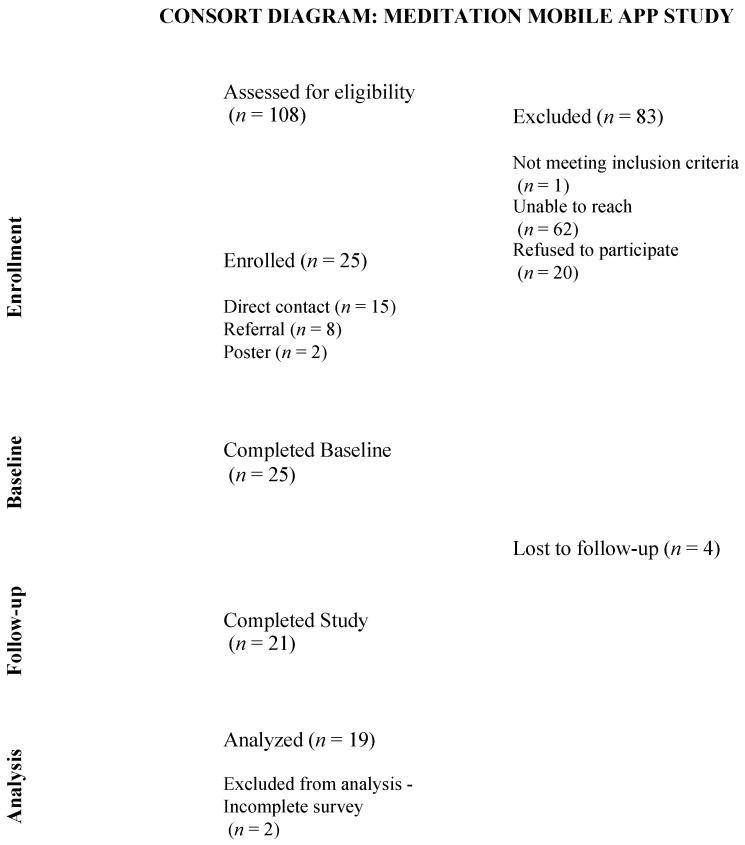
Consort Diagram.

A total of 108 patients were screened and only 1 person was not eligible. We were able to contact a total of 45 individuals who initially screened eligible (contact rate = 41.67%). Of those contacted, 20 declined to participate; 25 were enrolled in the study and completed the baseline survey (enrollment rate = 55.56%). Twenty-one participants completed the study (retention rate = 84%). We excluded *n* = 2 participants due to missing data (i.e., started but did not complete the post-survey), resulting in a total of 19 participants analyzed (see Figure 1).

**Figure 2:**
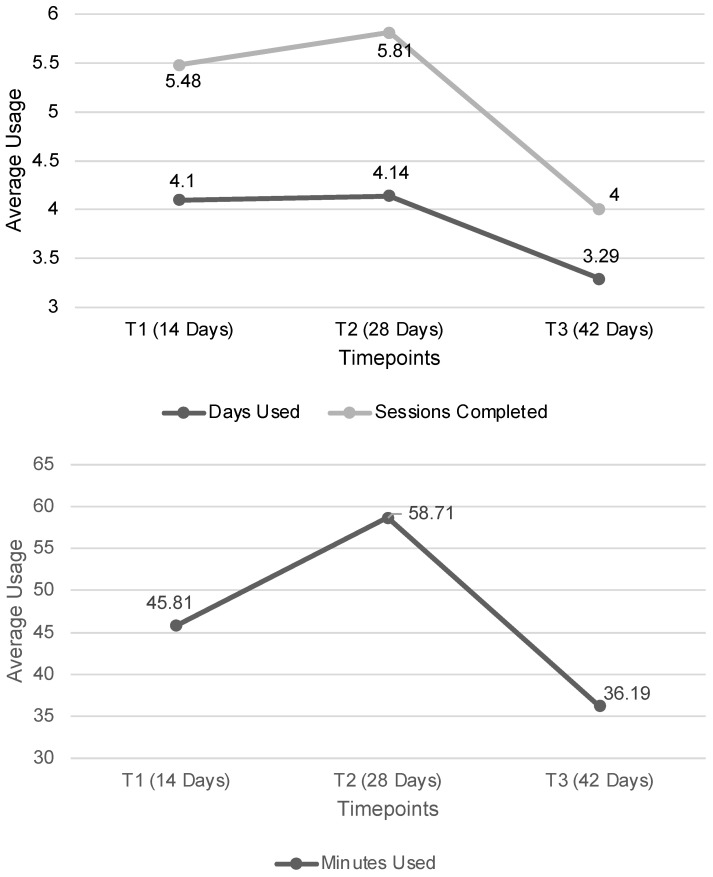
Duration of Ten Percent Happier Use.

User data were recorded at three time points (i.e., T1 = 14 days, T2 = 28 days, and T3 = 42 days). The average was 4.10 (*SD* = 3.30) for days used, 5.48 (*SD* = 4.33) for sessions completed, and 45.81 (*SD* = 40.54) for minutes used in the first 14-day period (T1). From T1 to T2, participants averaged 4.14 (*SD* = 4.85, *p* = 0.95) days used, 5.81 (*SD* = 8.24, *p* = 0.78) sessions completed, and 58.71 (*SD* = 90.73, *p* = 0.34) total minutes used. There were no significant differences between the T1 and T2 usages. However, there was a drop-off at T3; the average minutes dropped to 36.19 (*SD* = 12.85; *p* = 0.03). There were similar drop-offs in days and sessions used (M_Days_ = 3.29, *SD* = 0.93, *p* = 0.08; M_Sessions_ = 4.0, *SD* = 1.19, *p* = 0.08), but they were not significant (see Figure 2).

#### 3.2.2. Acceptability and Usability of Ten Percent Happier

On a 5-point scale, the uMARS app quality mean rating was 4.49 (*SD* = 0.32) and subscale mean ratings were 3.87 (*SD* = 0.63) for Engagement, 4.62 (*SD* = 0.42) for Functionality, 4.51 (*SD* = 0.45) for Aesthetics, and 4.48 (*SD* = 0.45) for Information Quality. The score for the subjective quality was 3.39 (*SD* = 0.80) and 4.21 (*SD* = 0.55) for perceived impact. Fourteen participants completed an exit interview discussing their experience related to the study and the app. Three core themes were identified in participants’ responses: (1) Participants’ Experience and Perceived Impacts, (2) Barriers and Potential Improvements, and (3) Dissemination. In Theme 1, most participants expressed positive experiences using the app, including aesthetics and functionality (e.g., easy to navigate). Lessons with multiple sessions were also very useful. Similarly, participants expressed that using the app to meditate often resulted in positive experiences which helped them use it more often. For example, participants found using the app to meditate before bedtime helped them relax and sleep better; Participant 1 shared “I usually used it in my apartment, usually toward the end of the day and in the evening, and it was kind of a nice way to wind down my day… I used it mostly as a winddown, decompression kind of tool”.

In Theme 2, participants spoke about some of the barriers and potential enhancements to the app. For example, many participants discussed how it was useful to develop a consistent schedule (e.g., using the app before bed daily). However, participants expressed that sometimes it was difficult to be consistent; if they missed multiple days, it would be very difficult to go back to meditating or using the app. For example, Participant 2 shared “Sometimes I would forget. But also sometimes when you just do it, you have obligations to do it. But generally, most of the time, if I didn’t do it for a day, I generally forgot about it”. However, the reminder notification function was somewhat helpful in alerting participants to keep using the app. Participant 10 shared “I also got these notifications on my phone… where in the evening around 11:00, just before I go to bed and it would pop up and say, ‘hey, don’t forget to do your meditation for today’. And sometimes I would see that and be like, ‘Oh, you know what, I haven’t done it today. Let me log in and do a session.’” Several participants suggested adding a social function with other peers; participants were interested in group mediation (both online or in-person) or online support groups. Participant 5 shared “Yeah, I would be interested [in social support on the app]. It would be cool to see others using the app. How they’re able to reap the benefits from the app. And it’s always good to have some sort of community, just some sort of bonding. So, it’s good to have some sort of community get-together and talk about the app, or just different stressors that have been relieved by the use of the app”.

Theme 3 described responses to the question about the best ways to share information about meditation mobile apps with other AYA cancer supports. Participants suggested using social media. Participant 12 shared “I think texting people is definitely [helpful]—it got my attention right away. I know the clinic sends mail to us, like with flyers. You could do that too”. Participant 5 also shared “There are some survivor groups on Facebook… Maybe if you were to put advertisements on those kinds of support groups as well… I think it’s reaching out to certain groups, whether it’s social media, like Instagram, Facebook…” Participants also suggested that they would trust information, including meditation mobile apps, if it came from their clinical team or if it was recommended on their clinic’s website. Participant 10 shared “I would say if my doctor and oncologist’s office suggested it and recommended it, that’s good enough for me”.

When asked if they would want to see cancer survivorship-related content, participants were interested and mentioned stress stems from their medical needs. Participant 24 shared “I think being a cancer survivor, I have work and I have work stress… But I also have very intense anxiety and things like that that are from my experience as a cancer survivor… Medical worries are something that as a cancer survivor, I am always gonna be dealing with. It would be interesting to have some sort of a series that [focuses] on handling stress and worries that come with medical concerns. [For example] worries about like you have a doctor’s appointment coming up”.

### 3.3. Changes in Outcomes after Using Ten Percent Happier

Table 2 presented the comparison of the outcomes between the baseline and post-treatment. At post-treatment, only Mindfulness was statistically significant (*p* = 0.001; *d* = 0.74). As a feasibility study, our focus was on examining effect sizes rather than statistical significance. Using Cohen’s *d* to estimate the treatment effects, Negative Emotion (*d* = 0.48), Perceived Stress (*d* = 0.52), and Mental Health (*d* = 0.45) resulted in medium improvements. 

### 3.4. Associations between User Engagement and Outcomes

We also explored potential relationships between app usage and reported outcomes. First, we conducted a series of correlations to explore the usage and mean difference between pre- and post-treatment. Only sleep disturbance was found to be associated with usage at T3 (*r* = −0.47 for day uses, *p* = 0.05; *r* = −0.48 for sessions, *p* = 0.04; *r* = −0.47 for minutes use, *p* = 0.05). Then, we separated the users by their app engagement; engaged users were defined by consistent app usage (i.e., having used the app in each of the three time points). We then used independent *t*-tests to compare the mean difference scores between engaged users and disengaged users. Engaged users reported higher mean difference scores in Mindfulness (*d* = 0.74), Perceived Stress (*d* = 0.83), Mental Health (*d* = 0.51), Meaning and Purpose (*d* = 0.84), and Sleep Disturbance (*d* = 0.81) compared to their disengaged counterparts (see Table 3).

## 4. Discussion

Results indicate that Ten Percent Happier is feasible and acceptable to use by AYA survivors of childhood cancer. Specifically, the relatively high retention rate demonstrated the feasibility of the Ten Percent Happier meditation mobile app intervention among this population. The user data gave us an early picture of how users engage with the intervention outside of the laboratory setting. For example, there was a steady decline in user engagement at T3, which was more than one month from the start of the study. This finding aligns with other mindfulness-based apps [42], which suggests a common issue among many meditation apps. Ten Percent Happier also demonstrated acceptability; it received a rating of at least a 4 rating (out of 5) across many categories on the uMARS except for Engagement (3.87) and Subjective Quality (3.39). This is particularly important because engagement with mHealth interventions is linked to intervention efficacy and generalizability [43,44]; studies have found a dose–response relationship with higher engagement linked to better treatment effects [45,46,47]. For AYA, there are many barriers to engaging in survivorship-related tools, including competing demands, such as work and school responsibilities, and psychosocial factors, such as health-related anxiety and avoidance [48]. Researchers should place emphasis on reducing app-related engagement barriers in a population that is already prone to disengagement. There is a potential need to incorporate components that motivate/reinforce usage (e.g., gamification [49] or human accountability support [50,51]).

The most commonly reported additional desired feature was the ability to socialize with other peers. Participants expressed interest in participating in group meditation (both online and in-person) or having a support group to share their experience with meditation and discuss other things. These suggestions align with the developmentally appropriate psychosocial need to socialize and connect with other peers who share a similar experience. Studies have found that meeting other AYA survivors has potential benefits including addressing areas of concern (e.g., late effects) with others with similar experiences [52], learning new coping skills, adopting positive health behaviors [53], and establishing meaningful relationships that promote psychological and social well-being [54,55]. To increase engagement, developers may consider including new features that foster these relationships between AYA survivors (e.g., online group meditation, the ability to connect with peers, and other social media capabilities).

The findings also suggested promising preliminary results in improving different patient-reported outcomes (i.e., mindfulness, negative emotion, perceived stress, and mental health). Moreover, we found that sustained usage was associated with improved outcomes. We found consistent engagement with the app resulted in better outcomes in Perceived Stress, Meaning and Purpose, and Sleep Disturbance compared with those who stopped using the app early. This aligns with the existing literature that shows a dose–response relationship of higher engagement linked to better treatment effects [45,46,47]. Researchers and developers should place emphasis on factors related to user engagement.

While this study has many strengths, including the use of a mixed-methodological approach and objective user data, there are also several limitations. As a pilot trial intended to assess intervention feasibility and acceptability to inform future research, the changes in outcome measures should be interpreted with caution. With a small sample size in a single-arm design, there was limited statistical power to detect modest treatment effects or make any generalizable inference (i.e., no comparison group). We also could not conduct any mediational analyses (e.g., meditation pathway: mindfulness → cognitive appraisal → outcomes, as stated in the Mindful Coping Model [56]) due to the small sample size. In addition, we did not assess whether participants were concurrently engaging in counseling/therapy or had prior experience with meditation/meditation apps; those with other experiences may be more likely to engage positively with this app. We also did not explicitly discourage participants from trying other meditation apps during the study period, though the qualitative data from exit interviews did not suggest that participants actively tried other apps during the study period. Further, it is possible that those who agreed to participate represent a self-selected group of individuals who are generally more interested in using a meditation app, which could bias results towards increased use and the reporting of more positive experiences. Having a comparison group and using random assignments is important for future work to address these potential biases. Future trials should also employ larger samples, include an active comparison condition, and extend the follow-up assessment period.

## 5. Conclusions

Despite these limitations, the current findings add to the literature on mHealth tools for AYA survivors of childhood cancer. Ten Percent Happier and other meditation mobile apps offer accessible, relatively low-cost coping tools that can reach large numbers of distressed survivors who may not want or have access to traditional therapy. These preliminary results open new research possibilities; future studies should evaluate intervention efficacy using a randomized controlled trial design. Demonstrating treatment efficacy encourages future studies to move along the translational science continuum. For example, given its relative ease of use, clinical providers (e.g., oncologists, nurses, psychologists, social workers, etc.) and clinics/cancer organizations may consider recommending evidence-based apps to patients and survivors who present with subclinical distress or for well-being. Furthermore, clinical providers may consider adopting these apps in their clinical settings. Meditation mobile apps are mostly automated with minimal assistance, it does not increase or interfere with providers’ workflows. These may be easy to ‘prescribe’ for patients who might benefit from mindfulness meditation.

## Figures and Tables

**Table 1:** Demographics and baseline clinical characteristics of the sample, (*n* = 25).

Characteristics	Mean (*SD*) or *n* (%)
Age at inclusion, mean (*SD*)	23.35 (3.64)
Female, *n* (%)	13 (52.0)
Race, *n* (%)	
Non-Hispanic White	14 (56.0)
Non-Hispanic Black/African American	5 (16.0)
Non-Hispanic Asian	5 (20.0)
Hispanic	2 (8.0)
Currently in School, *n* (%)	10 (40.0)
Education	
Completed high school (graduate or GED)	1 (4.35)
Some college, vocational, or training school	8 (34.78)
Associate degree (e.g., A.A. or A.D. degree)	1 (4.35)
4-year college degree (e.g., B.A. or B.S. degree)	11 (47.83)
Post-graduate education (e.g., M.A., M.S., J.D., M.D., Ph.D., etc.)	2 (8.70)
Employment, *n* (%)	
Student	8 (32.0)
Working Full-time	16 (64.0)
Unemployed	1 (4.0)
Household Income, *n* (%)	
Less than $50,000	3 (16.67)
$50,000 to $74,999	7 (38.89)
$75,000 to $99,999	5 (27.78)
More than $100,000 to $149,999	3 (16.67)
Living with, *n* (%)	
Parent(s)/Caregiver(s)	15 (60.0)
Sibling/Roommate	5 (20.0)
Spouse/Significant other	2 (8.0)
Live alone	3 (6.0)
Marital Status, *n* (%)	
Single	17 (68.0)
In a Relationship	8 (32.0)
Insurance, *n* (%)	
Employer/School	13 (52.0)
Parent	7 (28.0)
Medicaid	3 (12.0)
Unsure	2 (8.0)
Cancer Diagnosis, *n* (%)	
ALL	10 (40.0)
Hodgkin/Non-Hodgkin Lymphoma	8 (32.0)
Sarcoma	4 (16.0)
Other (i.e., AML and brain and spinal cord tumors)	3 (12.0)
Relapse, *n* (%)	4 (16.0)
Treatment Received, *n* (%)	
Chemotherapy	24 (96.0)
Radiation	6 (24.0)
Surgery	3 (12.0)
Transplant	2 (8.0)
Limb Salvage Surgery	2 (8.0)
Number of Late Effects, *n* (%)	
None	7 (28.0)
One	4 (16.0)
Two	4 (16.0)
Not Sure	8 (32.0)

**Table 2:** Preliminary Treatment Effects on Study Outcomes.

Measure	Baseline M (SD)	Posttreatment M (SD)	[95% CI]	*t*(df)	Cohen’s *d*
Mindfulness	3.55 (0.73)	4.08 (0.64)	[0.25, 0.82]	*t*(18) = 3.91 *	0.74
Cognitive Reappraisal	4.89 (1.13)	5.06 (0.88)	[−0.29, 0.64]	*t*(18) = 0.79	0.16
Positive Emotion	15.47 (0.83)	16.26 (4.24)	[−1.09, 2.67]	*t*(18) = 0.88	0.28
Negative Emotion	12.84 (3.34)	11.58 (3.19)	[−2.81, 0.28]	*t*(18) = −1.71	0.48
Perceived Stress	20.32 (5.41)	18.11 (6.37)	[−5.61, 1.19]	*t*(18) = −1.38	0.52
Physical Health	14.84 (3.13)	15.58 (3.09)	[−0.24, 1.71]	*t*(18) = 1.59	0.37
Mental Health	12.11 (3.36)	13.26 (3.60)	[−0.34, 2.66)	*t*(18) = 1.62	0.45
Meaning and Purpose	15.32 (4.30)	15.47 (3.66)	[−1.45, 1.77]	*t*(18) = 0.21	0.27
Sleep Disturbance	10.32 (4.32)	9.37 (4.63)	[−2.80, 0.91]	*t*(18) = −1.07	0.21

* *p* ≤ 0.05; Cohen’s *d*: small (*d* = 0.2), medium (*d* = 0.5), and large (*d* = 0.8) [41].

**Table 3:** Comparison of Mean Difference Scores between Engaged (*n* = 11) and Disengaged (*n* = 8) Users.

Measure	Engaged (*n* = 11) M (SD)	Disengaged (*n* = 8) M (SD)	[95% CI]	*t*(df)	Cohen’s *d*
Mindfulness	0.45 (0.46)	0.65 (0.76)	[−0.39, 0.79]	*t*(17) = 0.73	0.34
Cognitive Reappraisal	0.25 (0.33)	0.06 (0.28)	[−1.16, 0.77]	*t*(17) = −0.43	0.20
Positive Emotion	1.00 (3.41)	0.50 (4.72)	[−4.42, 3.42]	*t*(17) = −0.27	0.13
Negative Emotion	−1.73 (3.47)	−0.63 (2.92)	[−2.09, 4.29]	*t*(17) = 0.73	0.34
Perceived Stress	−4.55 (5.61)	1.00 (7.91)	[−0.98, 12.07]	*t*(17) = 1.79	0.83
Physical Health	0.82 (1.89)	0.63 (2.33)	[−2.23, 1.85]	*t*(17) = −0.20	0.09
Mental Health	1.82 (2.99)	0.25 (3.24)	[−4.61, 1.47)	*t*(17) = −1.09	0.51
Meaning and Purpose	1.27 (3.13)	−1.38 (3.16)	[−5.73, 0.43]	*t*(17) = −1.81	0.84
Sleep Disturbance	−2.40 (3.06)	0.63 (4.44)	[−0.72, 6.77]	*t*(17) = 1.71	0.81

Cohen’s *d*: small (*d* = 0.2), medium (*d* = 0.5), and large (*d* = 0.8) [41].

## Data Availability

The data that support the findings of this study are available from the corresponding author upon reasonable request. The data are not publicly available due to privacy or ethical restrictions.
